# Availability of Antidotes for Management of Acute Toxicity Cases at Emergency Departments in Qassim Hospitals: A Retrospective Study

**DOI:** 10.7759/cureus.28992

**Published:** 2022-09-09

**Authors:** Mahdi H Alsugoor

**Affiliations:** 1 Emergency Medical Services, College of Health Sciences–AlQunfudah, Umm Al-Qura University, Makkah Province, SAU

**Keywords:** toxicity, antidotes, emergency department, acute toxicity, drugs

## Abstract

Background: Drug overdose is a medico-social issue worldwide that may occur intentionally or unintentionally. It is one of the most common reasons for emergency department visits, and it is also a frequent cause of morbidity and mortality globally. This study aims to determine the occurrence of acute toxicity cases and their management outcomes at the emergency departments in Qassim Province hospitals in Saudi Arabia. In addition, the study aims to investigate the antidote availabilities at those medical centers.

Methods: A retrospective hospital record-based study of acute toxicity cases admitted to the emergency department in hospitals in Qassim during the period from January 1, 2020, to December 31, 2020, was conducted. Data were collected based on hospital resources such as gastrointestinal decontamination, stabilization, elimination enhancement resources, and antidotes from Qassim hospitals, and the availability of antidotes as well as the clinical data of the patients with the management outcome.

Results: A total of 264 patients with acute toxicity were admitted to the emergency departments of 14 hospitals in Qassim Province in 2020. Of the 264 cases, 179 (68%) were males, and 85 (32%) cases were females. Ninety-five percent of the cases were admitted to public hospitals, whereas 5% were admitted to private hospitals. The largest group by age of admitted cases were aged 11-20 years (19.3%). This study showed that 99% received appropriate treatment for their cause of toxicity, whereas 1% did not. The most common causes of toxicity in Qassim were found to be food poisoning (20.5%), followed by intentional suicide attempts with warfarin/enoxaparin/aspirin overdoses (15.9%) and acetaminophen (paracetamol) overdosage seen in 15.5% of admitted cases. Flagyl, in addition to fluids, was used in the management of 16.7% of cases, N-acetyl cysteine was used for 16.3%, and vitamins K and B6 were used for 14.0% of cases. Activated charcoal, atropine, calcium chloride, calcium gluconate, flumazenil, insulin, magnesium, sodium bicarbonate, and vitamin K were available at all the studied hospitals. However, all the hospitals lacked both ethylenediaminetetraacetic acid (EDTA) and a cyanide kit. Methylene blue and leucovorin were available in only one of the studied hospitals.

## Introduction

Drug overdose, or poisoning, is a global public health burden for both adults and children and is one of the most common reasons for admission to hospital emergency departments [1-4]. Intentional and accidental acute poisonings occur from a range of medicines, household products, and industrial chemicals and frequently need medical treatment, includ­ing hospital admission, worldwide. Accidental or unintentional poisoning is more common in children, whereas suicidal poisoning is more common in adults. The most common cause of intentional poisoning is insecticide self-poisoning, which accounts for about one in seven of the world's suicides [5].

The treatment of acute toxicity and poisoning cases in emergency departments starts with stabilizing the patient, evaluating the vital signs, initiating an airway, breathing, and circulation, followed by gastrointestinal (GI) decontamination, or using an antidote immediately [6-7]. Effective management of these poisoning cases is crucial for decreasing mortality and morbidity, especially if the common poisoning causes are adequately defined.

Comprehensive studies have been conducted to investigate acute toxicity causes and management. For example, a study conducted in North India investigated the profile, pattern, and outcome of oral poisoning cases admitted to the emergency department of a tertiary care teaching hospital. Records of poisoning cases have reviewed the kind of poison, the route of poison, and the outcome of treatment. Most of the poisoning cases belonged to rural areas (60.8%). The foremost commonly implicated agents were pesticides (75.3%), followed by drug overdose (20.52%) -- nearly 94% of cases recovered after treatment [8]. Another study in Palestine has analyzed the pattern of acute toxicity in patients admitted to government hospitals and found that more than 92% of patients were of the unintentional type of poisoning. The causative agents were mainly biological agents (77.4%), pharmaceuticals (11.6%), and other chemicals (10.9%). The most common routes of poisoning were through stings (72.3%) and then oral ingestion (23.5%) [9].

In Saudi Arabia, research conducted in 2020 studied the frequency and management of acute poisoning among children attending an emergency department at East Jeddah hospital. Results showed that unintentional poisoning of children occurred in 56.6% of observed cases. Medications were the most common cause of poisoning, accounting for 76.8%. More than 80% of patients showed oral ingestion as the most common route of poisoning [10].

In Western Saudi Arabia, a study has investigated the pattern of drug overdose and chemical poisoning among patients attending an emergency department. The most affected age group was children under the age of 12 (44.2%). The most typical type of poisoning was caused by drug overdose (92.2%). The majority of used medications were analgesics and non-steroidal anti-inflammatory drugs (20.4%). Central nervous system symptoms were the most frequently reported symptoms (57.4%), followed by gastrointestinal symptoms (41.9%). Thirty-four incidents of intentional poisoning were reported (26.4%). Compared to male patients, female patients attempted to commit suicide at a much higher rate [11].

As a result of the importance of the availability of antidotes and their potential direct effect on poisoning patterns, many national and international studies have investigated the availability of antidotes at hospitals and medical centres [12-16]. As highlighted earlier, acute toxicity is a national and international health issue that needs to be studied extensively to reduce its occurrence. The first step is for information regarding acute poisoning and patients' management outcomes to be investigated. In the present study, we aimed to explore the nature and severity of acute toxicity in Qassim Province and ensure the availability of the antidotes and medications required to undertake appropriate preventive and treatment measures. To the best of our knowledge, this is the first study to investigate the availability of antidotes in Qassim Province, Saudi Arabia.

## Materials and methods

Ethical considerations

Confidentiality of the patient's information was maintained. Prior to data collection, ethical approval was obtained from the biomedical research ethics committee, College of Medicine, Umm Al-Qura University. The ethical approval number is HAPO-02-K-012-2022-03-1001.

Study population and study period

This was a retrospective multicenter study that was conducted at the emergency medical departments of eligible hospitals in Qassim Province. Acute toxicity cases were presented to these hospitals during a one-year period from January 1, 2020, till December 31, 2020. Data collection took place by contacting the emergency department of the college inquiring about the availability of the antidotes, and the acute poisoning data were collected from the records of all hospital patients who visited the emergency departments during the specified period.

Sources of data for the availability of the antidotes stocking

All patients with acute poisoning who presented to and were managed within the emergency departments of 14 hospitals (13 public and one private hospital) in Qassim Province from January 1 to December 31, 2020, were reviewed. We employed a prestructured format that inquired about treatment records for patients who presented with acute toxicity in the emergency center. Data of the patients who were reviewed by a single reviewer of the college (blinded about the groups) included gender, age, season, time of the toxicity incident, causes of toxicity, type of toxicity, toxicity route, patient status, treatment, duration of hospital stay (if any), and outcome of the treatment. Regarding the availability of antidotes, the questionnaire was filled out by the health professionals responsible for stocking the antidotes within the emergency units. The questionnaire included questions on the hospital’s geographical location, whether it was in the general public or the private sector, the number of hospital beds, and the toxicity or poisoning-related information.

Data analysis

Collected data were analyzed using SPSS version 21.0 statistical software (IBM Corp., Armonk, NY). Descriptive statistics (frequency and percentages) were used to describe the categorical study and outcome variables.

## Results

A total number of 264 patients with acute toxicity were admitted to the emergency departments of 14 hospitals in Qassim Province during the calendar year 2020. Of the 264 cases, 179 (68%) were males and 85 (32%) were females. Ninety-five percent of the cases were admitted to public hospitals, and 5% were admitted to the participating private hospital. About 5.3% of the admitted patients were in the age group 1-10 years, 19.3% of the cases were aged 11-20 years, and 17.4% were in the 21-30 years age group. Around 89% of the acute poisoning cases were reported by hospitals located in cities, while the rest were in hospitals in villages (Table 1).

**Table 1:** Sociodemographic data of the acute toxicity cases treated in Qassim hospitals during 2020.

Age groups		Males no. (%)	Females no. (%)	Total no. (%)
1	1-10 years	9 (5%)	5 (5.9%)	14 (5.3%)
2	11-20 years	34 (19%)	17 (20%)	51 (19.3%)
3	21-30 years	32 (17.9%)	14 (16.5%)	46 (17.4%)
4	31-40 years	31 (17.3%)	9 (10.6%)	40 (15.2%)
5	41-50 years	28 (15.6%)	8 (9.4%)	36 (13.6%)
6	51-60 years	20 (11.2%)	12 (14.1%)	32 (12.1%)
7	61-70 years	16 (8.9%)	11 (12.9%)	27 (10.2%)
8	>70 years	9 (5%)	9 (10.6%)	18 (6.8%)
Number of acute toxicity cases treated in urban and rural hospitals, respectively				
1	Urban	236 (89.4%)		
2	Rural	28 (10.6%)		
Hospital sector-treated acute toxicity cases				
1	Public	250 (94.7%)		
2	Private	14 (5.3%)		

Regarding percentages of patients who received treatment in the hospitals, we found that 99% received appropriate treatment for their toxicity, whereas 1% did not.

**Figure 1:**
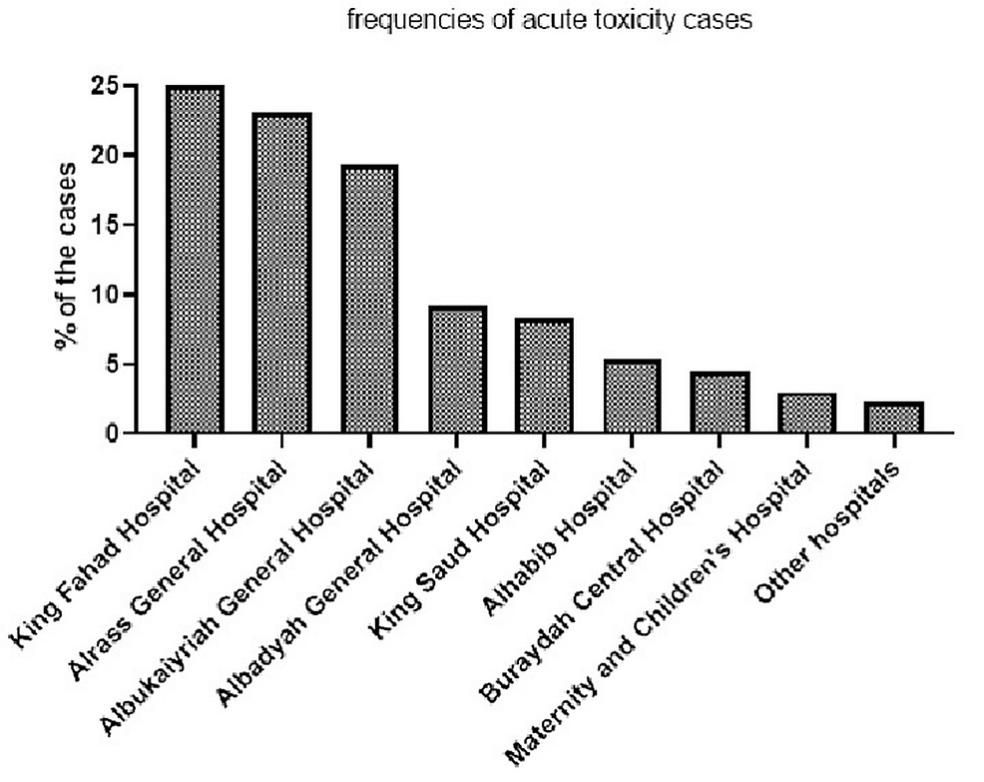
Percentage of the acute toxicity cases managed in the different emergency departments of Qassim hospitals.

Figure 1 shows the distribution of admissions of toxicity cases in various hospitals in Qassim Province. King Fahad hospital received 25% of toxicity cases, followed by Alrass General Hospital (23.1%) and Albukayriah General Hospital (19.3%).

**Figure 2:**
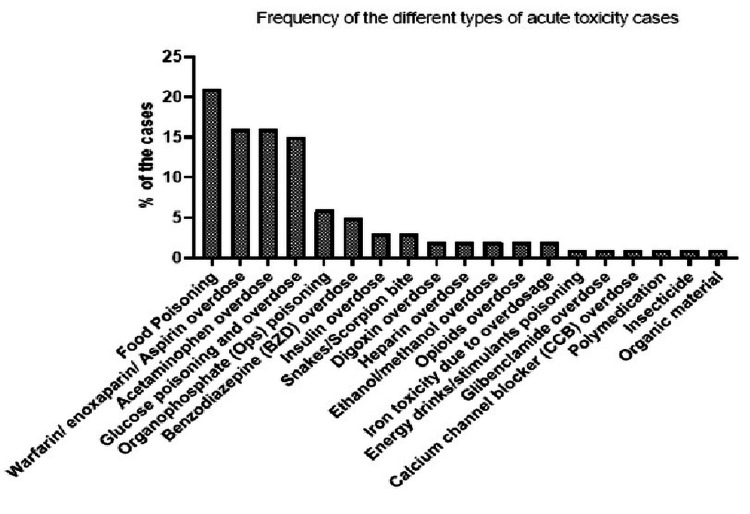
Frequency of the different types of acute toxicity cases diagnosed in the emergency departments of Qassim hospitals during 2020.

The most common cause of Qassim poisoning cases was food poisoning (20.5%), followed by warfarin/enoxaparin/aspirin overdoses (15.9%), acetaminophen (paracetamol) overdoses (15.5%), acute hyperglycemia (14.8%), and organophosphate (OP) poisoning (5.7%) (Figure 2).

The clinical symptoms presented during admission of the acute toxicity cases in emergency departments are shown in Table 2. Vomiting, diarrhea, and gastrointestinal disturbances were noted among 21.6%, bleeding was noted in 15.9%, and hyperglycemia and hypoglycemia were reported in 12.9% (Table 2).

**Table 2:** Frequencies of clinical symptoms presented by the acute poisoning cases at the time of admission to the emergency medical departments of Qassim hospitals. GIT, gastrointestinal tract

S. No.	Clinical symptoms presented during the admission	No. of cases (%)
1	Vomiting and diarrhea + GIT disturbance	57 (21.6%)
2	Bleeding	42 (15.9%)
3	Hyperglycemia/Hypoglycemia	34 (12.9%)
4	Liver impairment symptoms	27 (10.2%)
5	Confusion	20 (7.6%)
6	Abdominal pain	14 (5.3%)
7	Palpitations/tachycardia	13 (4.9%)
8	Deep sedation	9 (3.4%)
9	Coma	9 (3.4%)
10	Dizziness	7 (2.7%)
11	Elevated blood pressure/hypotension	4 (1.5%)
12	Increased saliva and tear production, constricted pupils, sweating, muscle tremors, and confusion	4 (1.5%)
13	Constipation	2 (0.8%)
14	Others	22 (8.3%)

**Figure 3:**
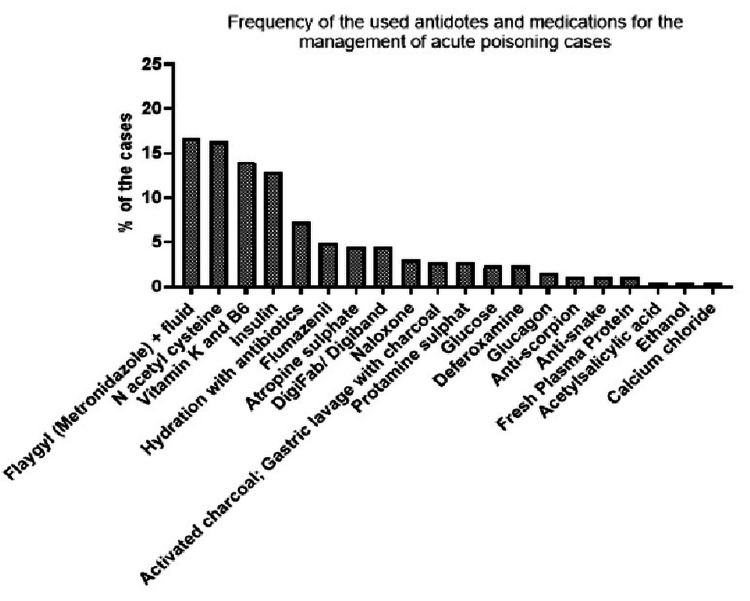
Frequency of the used antidotes and medications for the management of acute toxicity cases in the emergency departments of Qassim hospitals.

Figure 3 shows the management of acute toxicity cases who were admitted using different types of medications present in the emergency departments at the participating hospitals. Flagyl and fluids were used as a treatment to manage 16.7% of cases, N-acetyl cysteine was used for 16.3%, vitamins K and B6 were used in 14.0% of cases, insulin in 12.9%, hydration with antibiotics among 7.2% cases, and flumazenil among 4.9% cases.

More than 96% of the acute toxicity cases showed improvement in their systemic findings with the antidote treatment in the emergency department, whereas 3% did not show any improvement; hence they were referred to other centers for further management, and death was reported in one case (0.4%) who was presented with intentional warfarin overdose.

Table 3 presents the availability of the antidotes and medications used for the treatment of acute poisoning and overdoses of drugs at different Qassim hospitals. From the availability tables, activated charcoal, atropine, calcium chloride, calcium gluconate, flumazenil, insulin, magnesium, sodium bicarbonate, and vitamin K were available at all the studied hospitals. However, all the hospitals were without any EDTA or a cyanide kit. Methylene blue and leucovorin were available only in one of the studied hospitals. The reasons for unavailable antidotes were mainly due to their availability in a nearby hospital, which accounted for 60% of the hospital stores reports, while other hospitals replied that they have available alternative medications (40%).

**Table 3:** Availability of different antidotes and medications in participating Qassim hospitals during 2020. EDTA, ethylene diamine tetraacetic acid

No.	Name of antidote	Availability in hospitals no. (%)	No.	Name of antidote	Availability in hospitals no. (%)
1	Activated charcoal	14 (100)	18	Insulin	14 (100)
2	Atropine	14 (100)	91	Isoproterenol	7 (50)
3	Calcium chloride	14 (100)	20	Leucovorin	1 (7.1)
4	Calcium gluconate	14 (100)	21	Magnesium	14 (100)
5	Cholestyramine	5 (35.7)	22	Methylene blue	1 (7.1)
6	Cyanide kit	0 (0)	23	N-Acetylcysteine (NAC)	14 (100)
7	D_50_W	13 (92.9)	24	Naloxone	14 (100)
8	Deferoxamine	7 (50)	25	Octreotide	7 (50)
9	Digoxin immune F	10 (71.4)	26	Hydroxocobalamin	6 (42.9)
10	Dimercaprol	2 (14.3)	27	Physostigmine/Pyridostigmine	10 (71.4)
11	EDTA	0 (0)	28	Pralidoxime	9 (64.3)
12	Ethyl alcohol (ethanol)	3 (21.4)	29	Protamine sulfate	13 (92.9)
13	Flumazenil	14 (100)	30	Pyridoxine	8 (57.1)
14	Folic acid	11(78.6)	31	Sodium bicarbonate	14 (100)
15	Fomepizole	2 (14.3)	32	Sodium nitrite	6 (42.9)
16	Glucagon	10 (71.4)	33	Sodium thiosulfate	2 (14.3)
17	Glucose	8 (75.1)	34	Vitamin K	14 (100)

## Discussion

Acute toxicity is a common medico-social problem worldwide. It consumes not only valuable health service resources but also causes considerable morbidity and mortality [17-19]. Factors affect the toxicity outcomes, including the degree to which the mechanism of toxicity is understood, the speed at which the patient arrives at clinical care, and the availability of effective medical treatment.

Our study showed that toxicity was more frequently observed in younger age groups (11-20 years and 21-30 years) than in other age groups. These findings were similar to other national and international reports. The national study in Qassim also showed more frequent poisoning rates in the 5-19 years and 20-49 years, age groups, while groups younger and older than those ages showed fewer toxicity incidents. A Polish research study investigating the poison cases from 2009 to 2011 revealed that the predominant poisoning cases were in people from 11 to 25 years old [20-21]. This could be justified by observing that older people are more aware of danger than younger ones and that children are protected by their parents. Another justification could be that the 10-20 year age groups in Saudi Arabia represent a high percentage (around 24%) of the Kingdom’s total population [22]. The present study reported that the toxicity ratio of males to females was around 2:1. In contrast, various other studies have reported a high female preponderance [23-24]. This contradiction could result from those studies finding a dominant percentage of intentional poisoning rates, which was not the case in our study, and it is known that females are predominant in intentional cases [25].

Our data showed that the most frequent cause of toxicity in Qassim was food poisoning (20.5%), followed by warfarin/enoxaparin/aspirin overdose (15.9%), and acetaminophen (paracetamol) overdoses that were seen in 15.5% of admitted cases. A similar trend was also shown in a local study where food poisoning was the highest cause of poisoning for the cases admitted to the emergency departments [20]. In contrast, the causes of toxicity vary with location and country with the different accessibility to the chemical compounds. One study showed the main reasons for toxicity were due to pesticides (39.5%) followed by medicines (26.1%) [26]. Maheswari et al. (2016) reported that drug toxicity was the main reason given at the admitting care unit; different pharmacological groups were involved; psychiatric drugs were used by 58.9%, followed by levothyroxine 7.7%, analgesics 12.8%, antihypertensives,7.7%, and unknown drugs 12.8% [27], which was consistent with a study conducted by Jalali et al. (2012), where 31.6% of cases were due to consumption of medicines such as antidepressants (58%) followed by antihypertensives (11.4%) [8].

Accidental poisoning was reported to represent 5.3% of the cases in our study, and the rate was higher in children aged between 1 and 10. Similarly, Maheswari et al. (2016) showed that accidental poisoning (8.91%) was more frequent in children aged between 1 and 10 years compared to 0.99% of suicidal cases [27]. Children's accessibility to poison is influenced by socio-economic status, mothers' education level, family members' addiction, local beliefs, and customs of the community [28-30].

In our study, vomiting, diarrhea, and gastrointestinal disturbances were the main symptoms of the cases accounting for 21.6% of the total. This was followed by bleeding, hyperglycemia, and hypoglycemia. A similar trend was reported in a study that found that the majority of the patients presented with acute toxicity suffering from gastrointestinal complaints [31], which was different from another study where altered mental states were dominant [32]. This could be explained by the finding that the dominant cases of our study were caused by food poisoning, which is mainly linked with gastrointestinal symptoms.

Medications and antidotes used to manage toxicity cases were mainly metronidazole (Flagyl), N-acetylcysteine, and vitamins k and B6. These could be linked with the causes of patient toxicity mentioned earlier, as metronidazole could be used in food poisoning, N-acetylcysteine is used for paracetamol toxicity, and vitamins K and B6 are commonly used as antidotes for warfarin and isoniazid toxicity, respectively.

Our study showed a deep stock of medications and antidotes in the emergency departments: activated charcoal, atropine, calcium chloride, calcium gluconate, flumazenil, insulin, magnesium, sodium bicarbonate, and vitamin K were available at all the studied hospitals. However, all the hospitals lacked any EDTA and a cyanide kit, while methylene blue and leucovorin were available only in one of the studied hospitals. EDTA is a chelating agent used alone or concomitantly with dimercaprol for treating lead toxicity [33]. However, EDTA is not available in any of these hospitals, while dimercaprol is available in only two hospitals. Although there was no lead toxicity in our cases, the antidotes should be available at the hospital to counteract the poisoning quickly. Lead toxicity could occur from the use of lead-based paint in homes, in leaded petrol, or in other sources [34-35]. The Saudi Arabian government has prohibited all products containing lead [36]. This could explain the lack of lead toxicity in our data.

Our data showed a lack of a cyanide kit and inadequate stock of methylene blue which is used mainly for cyanide and carbon monoxide poisoning [37]. However, hydroxocobalamin and oxygen support could be used for cyanide toxicity and carbon monoxide toxicity, respectively [38]. Unfortunately, hydroxocobalamin was available in only 42.9% of the hospitals. Despite the lack of cyanide toxicity in our cases, ensuring the stock is available is crucial for a potential cyanide poisoning case.

Leucovorin reduces the effects of methotrexate. Our study reveals that leucovorin is available in only one hospital that specializes in cancer treatment where methotrexate is commonly used in such a hospital. However, methotrexate is also used in chronic inflammation such as arthritis [39]. Thus, not only the cancer specialized hospital has to stock leucovorin, but all other hospital should do as well.

Out of 264 patients included in our study, 263 (99.6%) improved, while one patient (0.4%) died. The cause of death was an intentional warfarin overdose. Despite the high percentage of patients’ recoveries, the medications and antidotes should all be available to counteract toxicity immediately and enhance patient outcomes.

It is vital that poisoned patients be referred to medical centers with adequate toxicological centers. Improved guidelines for antidote accessibility need to be developed and made available to emergency departments. Our study findings will help adopt effective measures for optimal stocking of antidotes and provide important implications for healthcare institutions and pharmaceutical practices. National practice guidelines are needed to be developed to assist emergency care in selecting appropriate antidotes based on the local pattern of poisoning incidents based on the similar types of studies conducted in all provinces in Saudi Arabia.

The weakness of the study is that any incompletely filled registry of poisoning cases, their management, and their outcomes in emergency departments were excluded from the analysis, and there is a greater chance of underestimating the incidence of acute poisoning as well as its outcomes with antidote availability.

## Conclusions

This study evaluated the prevalence of acute toxicity incidents in Qassim hospitals and the availability of antidotes and medications. The most common causes of acute toxicity among the admitted cases in Qassim hospitals were food poisoning, warfarin/enoxaparin/aspirin overdose, and acetaminophen (paracetamol) overdoses. Flagyl, fluids, and vitamin K were used in the management of nearly half the cases. Regarding the availability of the antidotes and medicines, activated charcoal, atropine, calcium chloride, calcium gluconate, flumazenil, insulin, magnesium, sodium bicarbonate, and vitamin K were available at all the participating Qassim hospitals. However, all hospitals lacked EDTA and none had a cyanide kit, while methylene blue and leucovorin were available only in one of the studied hospitals.

Hospitals must reassess their current antidote inventories and stock the specific antidotes that correspond with frequently observed poisonings. Therefore, healthcare policies that include specific guidelines must be developed and consistently adopted to cope with the standard practice within Saudi Arabia.
